# Accurate diagnosis of atopic dermatitis by combining transcriptome and microbiota data with supervised machine learning

**DOI:** 10.1038/s41598-021-04373-7

**Published:** 2022-01-07

**Authors:** Ziyuan Jiang, Jiajin Li, Nahyun Kong, Jeong-Hyun Kim, Bong-Soo Kim, Min-Jung Lee, Yoon Mee Park, So-Yeon Lee, Soo-Jong Hong, Jae Hoon Sul

**Affiliations:** 1 Department of Automation, Tsinghua University, Beijing, 100084 China; 2 Department of Human Genetics, David Geffen School of Medicine, University of California, Los Angeles, Los Angeles, CA 90095 USA; 3 Department of Biological Sciences, Korea Advanced Institute of Science and Technology, Daejeon, Daejeon, 34141 Republic of Korea; 4 Department of Medicine, University of Ulsan College of Medicine, Seoul, 05505 Republic of Korea; 5 Department of Life Science, Multidisciplinary Genome Institute, Hallym University, Chuncheon, 24252 Republic of Korea; 6 Department of Pediatrics, Asan Medical Center, Childhood Asthma Atopy Center, Humidifier Disinfectant Health Center, University of Ulsan College of Medicine, Seoul, 05505 Republic of Korea; 7 Department of Psychiatry and Biobehavioral Sciences, University of California, Los Angeles, Los Angeles, CA 90095 USA

**Keywords:** RNA sequencing, Metagenomics, Microbiome, Machine learning, Predictive medicine, Diagnostic markers, Predictive markers, Skin diseases

## Abstract

Atopic dermatitis (AD) is a common skin disease in childhood whose diagnosis requires expertise in dermatology. Recent studies have indicated that host genes–microbial interactions in the gut contribute to human diseases including AD. We sought to develop an accurate and automated pipeline for AD diagnosis based on transcriptome and microbiota data. Using these data of 161 subjects including AD patients and healthy controls, we trained a machine learning classifier to predict the risk of AD. We found that the classifier could accurately differentiate subjects with AD and healthy individuals based on the omics data with an average F1-score of 0.84. With this classifier, we also identified a set of 35 genes and 50 microbiota features that are predictive for AD. Among the selected features, we discovered at least three genes and three microorganisms directly or indirectly associated with AD. Although further replications in other cohorts are needed, our findings suggest that these genes and microbiota features may provide novel biological insights and may be developed into useful biomarkers of AD prediction.

## Introduction

Atopic dermatitis (AD) is a type of inflammatory skin disease resulting in red, itchy, swollen, cracked, and irritated skin, which is a severe form of eczema^1^. It is more prevalent in children but can occur at any age. Studies found that about 15% of children under 15 years of age are affected by AD in the United States^2^, while only about 7% of adults have AD^3^. In addition to the discomfort in the skin, children with AD may develop inhalant allergic diseases such as asthma^4^ and allergic rhinitis^5^ as well as mental disorders such as anxiety and depression^6^. Hence, AD may impose a high economic burden and have considerable negative effects on life quality^7–9^, which is a significant cost to society. However, there is no cure for this disease except a few treatments to relieve the symptoms^8^ because its causes are complicated^10^. For decades, treatment of AD has been limited to topical corticosteroids, topical calcineurin inhibitors, and for those with moderate to severe AD, systemic immunosuppressants^11^. In patients with moderate to severe AD, biological therapy such as dupilumab shows good results, but the high drug cost is a burden on patients with AD^10,11^.

Recently, the important role of colonic epithelial cells (colonocytes) has been implicated in the host–microbial interactions, and these gut epithelial cells contribute to the microbiota composition and activities following gut dysbiosis, affecting many chronic human diseases^12^. In addition, integration and correlation analyses of host genes expression and gut microbiota have emerged as an important opportunity for diagnosis and prediction of human diseases including AD^13,14^; for instance, associations between enzyme commission genes and microbiota in inflammatory bowel diseases^15^, and also between *IL-17* and *Streptococcus* in AD^16^. However, there have been few studies on prediction analysis using machine learning based on the gut transcriptome and microbiota in AD.

It is challenging to diagnose AD because of its variable morphology, distribution, and irregularity. Based on its main clinical features, diagnostic criteria have been developed and used worldwide^17,18^. Besides, the assessment of disease severity is problematic due to the lack of objective and effective markers^19^. This is concerning as physicians need to make decisions about treatment based on the diagnosis of AD and its severity, which might be related to the prognosis. Therefore, an accurate and automated diagnosis of AD and an improved set of biomarkers for it could have a potentially high impact.

In this paper, we develop a machine learning classifier for accurate and automated diagnosis for AD using the transcriptome of gut epithelial colonocytes and gut microbiota data. A classifier is an algorithm that implements classification, which maps the input data to specific classes. In our study, an AD classifier takes transcriptome data and/or microbiota data as input data and outputs the predicted status of AD. Specifically, we use transcriptome and metagenome data to achieve the comprehensive gene expression and microbiota profiles of individuals with moderate to severe AD and controls. We develop a robust machine learning pipeline including feature selection, model selection, cross-validation, classification, and follow-up statistical analyses, which can differentiate between subjects with and without AD based on the omics data with high accuracy.

## Materials and methods

### Sample collection and disease diagnosis

In this study, we collected the transcriptome of gut epithelial colonocytes and gut microbiota data of 161 subjects including 88 cases (patients with AD), 73 controls (healthy individuals). AD subjects were recruited from the Childhood Asthma Atopy Center of Asan Medical Center, Seoul, Korea, and were diagnosed in accordance with the criteria of Hanifin and Rajka^20^. All individuals are children aged from 6 to 72 months. The SCOring AD (SCORAD) value, as an important AD assessment index for the extent and severity of AD, was assessed by pediatric allergists based on the guidelines for the SCORAD index^21^. Total serum immunoglobulin (IgE) levels in the peripheral blood were measured using the ImmunoCAP system (Phadia AB, Uppsala, Sweden). The parents and guardians of all children provided written informed consent for their participation, and this study protocol was approved by the human ethics committee at Asan Medical Center (Institutional Review Board No. 2008-0616, 2015-1031, and 2017-0923).

### Transcriptome and microbiota data

Transcriptome data was obtained from mRNAs extracted the exfoliated colonocytes of each fecal specimen using the GeneChip Human Gene 2.0 ST Array (Affymetrix, Santa Clara, CA) under the manufacturer’s protocol. Microbiota data was obtained from the fecal samples using the Power Microbial RNA/DNA Isolation kit (MO BIO/Qiagen, Carlsbad, CA, USA), polymerase chain reaction (PCR) amplification based on primers targeting the V1-V3 variable region of 16S rRNA gene, and sequencing the Roche/454 FLX Titanium system (Roche, Mannheim, Germany) and MiSeq (Illumina, San Diego, CA) under the manufacturer's instructions. Since there was the requirement of actual read counts for quality control and the difficulty in a direct comparison between these two sequencing platforms, we focused on the common phylum and genus. More detailed information on the sequencing method is provided in our previous studies^22,23^.

### AD machine learning classifier

We built the supervised machine learning pipeline that predicts atopic dermatitis status using transcriptome and microbiota data. This pipeline includes prepossessing, feature selection, model selection and improvements, integration of microbiota data, and performance evaluation^24^. The pipeline is implemented with Python 3.7 and the scikit-learn package^25^.

#### Prepossessing

Initially, there were 161 samples in the transcriptome dataset and the microbiota dataset, respectively. 160 samples existed in both datasets. For one individual with only transcriptome data, we imputed its microbiota data using a mean values imputation approach that assumes missing values are missing completely at random (MCAR). For one individual with only microbiota data, we removed this sample as it is difficult to impute its transcriptome data due to a large number of genes to impute (one hundred times more genes in the transcriptome data than microorganisms in the microbiota data). At the end of this process, we have the set of identical 161 samples in both transcriptome and microbiota datasets.

Among the 161 samples, there were 88 AD patients and 73 controls, and we split them into the training set (*n* = 131) and the test set (*n* = 30). As the numbers of cases and controls were different, we used a stratified split to guarantee that the balance of cases and controls is consistent across training and test sets. We then used min-max normalization to scale the transcriptome and microbiota data so that the data range from 0 to 1, calculated as:

$${x}^{^{\prime}}=\frac{x-min(x)}{max(x)-min(x)}$$

where $x$ is a vector of the values of a feature. We use this normalization method because we want to ensure that the scaled data are positive. We normalize the training and test sets together.

#### Feature selection

As it is unlikely that a disease is strongly associated with more than 40,000 genes, most of the genes would be unrelated to the disease or have negligible effects. Therefore, feature selection on the training dataset is necessary to identify a subset of predictive genes, whose expression data could predict atopic dermatitis as accurately as possible. The two main aspects we considered were: (1) the optimal number of features to be selected in the entire dataset, and (2) the exact features chosen from the original training set.

Typically, there are three types of widely used feature selection methods. They are Filter, Wrapper, and Embedded methods^26^. We selected three methods from each type: Chi-squared Test, Recursive Feature Elimination (RFE), and Random Forest Classifier (RFC) because they are efficient and applied in the previous research^27^. Specifically, RFE requires the results from the models, and hence we chose Logistic Regression (LR), Support Vector Machine (SVM), and Random Forest Classifier (RFC) as the models in conjunction with RFE. These combinations are referred to as LR-RFE, SVM-RFE, and RFC-RFE, respectively.

After introducing the specific methods for feature selection, we should then consider the problem of overfitting. It is hard to extract correct features from high-dimensional datasets with small sample sizes.

Cross-validation (CV) is important in preventing overfitting^28,29^. In our task, we designed two plans (Plans A and B) for feature selection using cross-validation. Note that we do cross-validation and feature selection on the training set only so it will not cause data leakage. Plan A: we performed a 5 by 5 nested cross-validation for feature selection, which consisted of a fivefold inner CV round and a fivefold outer CV round. We used the outer CV on the entire training set to evaluate the model performance, and the inner CV is applied to the outer CV training split to select the set of predictive features (Supplementary Fig. S1A). In other words, supposing that the outer CV training split named *D* is used for feature selection, we executed a fivefold CV on *D* (i.e., inner CV) and determined the optimal number of features to select in *D*, which could achieve best average performance in the inner CV. Then we calculated the overlapping features across all training splits of the outer CV. Denote the number of features in the final set as *n*_*A*_. In a given inner CV training split, all the features are ranked by their weights (feature importance) assigned by the classification model trained with the inner CV training split. Then we selected top *k* features with *k* starting from 44,608 (all features) and being reduced by 10% in each iteration until *k* = 1, and trained models with the inner CV training split and evaluated them with the inner CV test split (the validation set). The optimal value of *k* was selected to generate the model with the best performance. In the outer CV training split, all the features are ranked using the same method as applied to the inner CV training split. Then we selected *k* top features to identify the set of predictive features for this outer CV training split, where *k* is the optimal number of features determined in the inner CV. The outer CV test set will be used for model selection and hyper-parameter tuning with the *k* selected features in the follow-up analysis instead of feature selection. We then repeated this process over all the five outer CV training splits and yielded five sets of predictive features. Finally, we selected the intersection set of them as the final set of predictive features for the entire original training set. Plan B: instead of using a nested cross-validation, we only performed one fivefold cross-validation on the entire training set and directly selected *n*_*B*_ top features on its training splits, *D* (Supplementary Fig. S1B, S2), where the features are ranked by test scores such as the p values of ${\chi }^{2}$ test between the features and the disease statuses. *n*_*B*_ is determined as the value that produces the model with the best average performance in the outer CV test sets (the validation set). The final set of predictive features are the top *n*_*B*_ features chosen in the entire original training set.

As mentioned before, to decide the two main aspects regarding feature selection, we considered different feature selection methods and many possible numbers of features based on a set of criteria. We used fivefold cross-validation to evaluate the performance. In detail, we employed the average F1-scores from outer CV test sets as metrics. We compared plan A and plan B, combined with five candidate feature selection methods: RFE-LR, RFE-SVM, RFE-RFC, Chi-square Test, and RFC. Based on the comparison results (see supplementary methods), we took the following steps to select the best feature number: we first calculated feature importance in each training split in the outer CV, ranked the features by their average feature importance, and chose the top *n* features from the training set whose feature importance was greater than a threshold (Plan B). Finally, we chose 35 features using the Chi-squared test in the entire original training set.

#### Model selection and improvements

We trained four different machine learning models, (1) Logistic Regression (LR), (2) Support Vector Machine (SVM) with linear and rbf kernels, (3) Random Forest Classifier (RFC)^30^, and (4) XGBoost^31^ with the outer CV training splits. XGBoost is a tree boosting method, demonstrated to perform extremely well in multiple classification tasks. We chose the best model among the four models by comparing the average F1-scores on outer CV test splits.

#### Jittering

Jittering is a useful tool to mitigate overfitting^32^. We added random noises to the training set of the original data before normalization and feature selection. The noise followed a normal distribution of

$$s\sim N(0, {\sigma }^{2}I)$$

where *I* is the intensity of noises and the variance ${\sigma }^{2}=1$. Although jittering might reduce the classification accuracy of the model on training sets, proper noises could increase the robustness of the algorithm, narrowing the performance gap between training and test sets, and therefore reducing the possibility of overfitting. Note that jittering is only performed on the transcriptome data in the training set. It is because the microbiota data contain many zero values and thus adding noises to it will distort the data.

#### Thresholding

Moreover, we consider the effect of changing the probability threshold (*p*_*t*_) in prediction. A sample is predicted to be a case by the model if the predicted probability is greater than a certain threshold (*p*_*t*_) where the default value is 0.5. Different probability thresholds should be examined to see whether they could further improve the model performance. For this improvement, we first selected the model with the best performance using the default probability threshold (0.5). Then we changed the machine learning model such that it generates the probability (*p*_*t*_) as output. We tested different thresholds and chose the best one based on the outer CV test split evaluation.

#### Feature importance

After comparison, we selected the Chi-squared test as our feature selection method. We used the “SelectedKBest" function in the scikit-learn package^25^ to implement the Chi-squared test. After identifying *k* features, we also want to rank the features based on a certain criterion. This function has an attribute named “*scores_*", and it returns the scores of features. The Chi-squared test is used to test the independence of two events.

In our binary classification problem, we have *X* as the input data with the size of (n_sample, n_feature), which are the number of samples and features respectively, and also *y* as the label of each sample with the size of n_sample. For calculation, we expand the size of *y* to (n_sample, 2). The first column of y represents the first class and the second stands for the second class. For each row, the elements will either be (1, 0) or (0, 1), which indicates that the sample of this row belongs to the first class or the second class.

After that, we calculated the observed result *f*_*obs*_,

$${f}_{obs}={y}^{T}X$$

Next, we calculated the expected result ${f}_{exp}$. To do this, first, we acquired feature_count, which is a (1, n_feature) matrix, and each column is the sum of this feature in each sample. Secondly, we obtained class_prob, which is a (1, 2) matrix, and each column is the mean of this class. Now we can get ${f}_{exp}$,

$$ f_{exp} = class\_prob^{T} \cdot \, feature\_count $$

Finally, we calculated the ${\chi }^{2}$ value by the following equation,

$${\chi }^{2}=\frac{{\left({f}_{obs}-{f}_{exp}\right)}^{2}}{{f}_{exp}}$$

where ${\chi }^{2}$ is a (2, n_feature) matrix. We summed up the result along the column and calculated the *scores_* vector of size n_feature. It represents the scores of the features, where column *i* of *scores_* is the score of the *i*-th feature.

In our task, higher values suggest higher importance of features. Therefore, we could compare each feature relatively from their feature importance.

#### Integration of microbiota data

In addition to the transcriptome data, we integrated the microbiota data to improve the performance of the AD classifier. We tested four methods when incorporating the microbiota data and evaluated their performance using the outer CV test set:

(1) a method that uses microbiota data only, (2) a method that uses transcriptome data only, (3) a method that combines transcriptome and microbiota data first, then performs feature selection and trains the model, and (4) a method that performs feature selection on transcriptome and microbiota data separately, then combines the two types of data and trains the model. We used similar feature selection methods as described above for microbiota data. The comparison of these four methods is in Supplementary Table S1.

#### Performance evaluation

We evaluated the prediction accuracy of the AD classifier using the test set, which is not used in training. We calculated several performance metrics including accuracy, precision, recall, and F1-score. In binary classification problems, we calculated those metrics as follows:

**Table:** 

Predicted class		
**Actual class**		
	True	False
True	*a *(True Positive)	*b *(False Negative)
False	*c *(False Positive)	*d *(True Negative)

$$\mathrm{Precision}=p=\frac{a}{a+c}, \mathrm{Recall}=r=\frac{a}{a+b} ,\mathrm{ Accuracy}=\frac{a+d}{a+b+c+d},\mathrm{ F}1-\mathrm{score}= \frac{2pr}{p+r}=\frac{2a}{2a+b+c}$$

We used F1-score as the main evaluation metric in this paper as it is a harmonic mean of precision and recall, leading to a more general and reliable assessment of the model performance, especially when classes are imbalanced. F1-score ranges from 0 to 1 where the performance is better when the F1-score is closer to 1. In addition to these metrics, we plotted the Receiver Operating Characteristic (ROC) curve by plotting the true positive rate (TPR) against the false positive rate (FPR) at different threshold settings. We then calculated the area under the ROC curve (AUC), which also ranges from 0 to 1 where 1 represents the perfect performance.

#### Assumptions in experimental settings

In building and testing the AD classifier pipeline, we have several hyper-parameters such as feature numbers, feature selection methods, and training models. As examining all combinations of hyper-parameters is exponential in the number of hyper-parameters, our experiments are based on the assumption that every variable or hyper-parameter is weakly correlated to each other. It means that optimizing a hyper-parameter one at a time yields a similar model when optimizing all hyper-parameters at the same time.

### Ethics approval

The parents and guardians of all subjects provided written informed consent for their participation, and this study protocol was approved by the human ethics committee at Asan Medical Center (Institutional Review Board No. 2008-0616, 2015-1031, and 2017-0923). All research was performed in accordance with relevant guidelines and regulations.

## Results

### Data description

We acquired the transcriptome profiles and the microbiota data of 161 subjects, who were recruited from the Cohort for Childhood Origin of Asthma and Allergic Diseases birth cohort and the Asthma Atopy Center of Asan Medical Center, Seoul, Korea. After preprocessing, there are 88 cases (patients with AD) and 73 controls (healthy individuals). As summarized in Table 1, the mean age was higher in the AD patient group than the controls (17.37 ± 3.48 month vs. 10.81 ± 2.15 month, *P* = 0.001), and the serum total IgE levels were significantly higher in the AD group (243.06 ± 160.21 IU/ml vs. 22.83 ± 9.46 IU/ml, *P* = 0.004). On average, subjects without AD are 3.16 months younger than individuals with the disease. After preprocessing and normalizing the raw gene expression and the microbiota data, there are 44,608 gene expression probes and 366 taxa of microorganisms used for developing a machine learning pipeline.

**Table 1:** Baseline characteristics of the subjects in this study.

	All	Cases (AD)	Control (No AD)	Cases (AD) vs control (No AD) t test p value
Average age: months	14.21 ± 2.14	17.37 ± 3.48	10.81 ± 2.15	0.001
Sex: female	72	32	40	–
^a^SCORAD	–	32.86 ± 5.49	–	–
Total IgE (IU/ml)	135.191 ± 83.53	243.06 ± 160.21	22.83 ± 9.46	0.004

^a^SCORAD: SCOring AD value, an AD assessment index that is only available for patients.

### Developing atopic dermatitis classifiers

**Figure 1:**
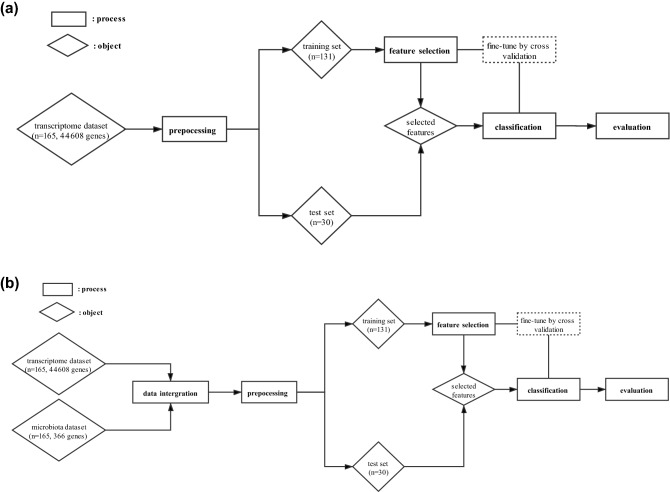
The overview of atopic dermatitis classification pipelines in two settings. (**a**) Transcriptome dataset only, and (**b**) transcriptome and microbiota data.

To accurately predict AD incidence, we design two machine learning classifiers: one using only transcriptome data (Fig.1a) and the other using both transcriptome and microbiota data (Fig.1b). Both classifiers consist of several computational steps, and we describe each step briefly here (see “Materials and methods” for details). First, we preprocess the data such as removing duplicates, imputing missing values, splitting the data into training and test sets, and performing normalization. Second, we perform feature selection using the training set with the cross-validation to identify the best set of features for prediction (e.g., expression of specific genes or specific taxa) as well as to choose the best machine learning model. To improve the performance of the classifier, we consider changing a few hyper-parameters such as adding certain levels of noise to expression data and changing the probability threshold to classify cases and controls. Lastly, we apply the trained machine learning model and selected features to the test set for classification and evaluate the performance of the machine learning model. As our dataset consists of a high-dimensional feature set from a limited sample size, we primarily focus on developing a machine learning classifier that is robust with the small sample size, prevents overfitting, and prioritizes genes or features for prediction.

### Evaluation of transcriptome only classifier

The transcriptome data are available from 161 individuals whose gene expression profile is measured at 44,608 probes (“features”). As a large number of features have negative effects on classification performance such as causing overfitting, we perform feature selection to identify a subset of informative features. We use a fivefold cross-validation approach using a training set (n = 131) and test several feature selection methods such as recursive feature elimination (RFE), support vector machine (SVM), and chi-squared test. We measure the performance of feature selection methods using F1 score and find that the chi-squared test approach selecting about 35 features from the training set has the best performance. So we decide to select 35 features with feature importance $\ge$ 0.95 which can achieve the highest performance (Supplementary Fig. S3).

Once we identify the set of best features or genes for prediction, we next seek to identify the best machine learning model for prediction. We test several machine learning (ML) models such as Logistic Regression (LR), Support Vector Machine (SVM), Random Forest Classifier (RFC), and XGBoost. We use a 5-fold cross-validation with a training set and 35 features to evaluate the performance of each ML model and find that SVM with the rbf kernel has the highest F1 score (Supplementary Table S2). To improve the performance of our ML classifier, we vary the probability threshold (*p*_*t*_) when making predictions on the case-control status; an individual is predicted to be a case if the predicted probability is greater than *p*_*t*_ and by default, *p*_*t*_ = 0.5. Results show that *p*_*t*_ = 0.3 generates the best F1 score using 5-fold cross-validation (Supplementary Table S3). Lastly, another improvement we make to the ML classifier is jittering, which is adding random noises to transcriptome data. With jittering, it may be difficult for the machine learning models to fit the data, and therefore it may enhance the generalization ability and reduce the overfitting. We add different levels of noise and observe the highest F1 score with a noise level of *I* = 0*.*001 using SVM on 5-fold cross-validation (Supplementary Table S4).

After we identify the best ML model and features as well as improvements based on the performance using the training set, we evaluate the AD classifier on the test set (n = 30). We use a variety of metrics such as F1 score, accuracy, precision, recall, and the area under the curve (AUC) under the receiver operating characteristic (ROC) curve. In addition to the best AD classifier we identified, we also test classifiers without feature selection and the improvements to observe their impact on the performance. Specifically, we test four models: (1) SVM with all features, (2) SVM with the best 35 features, (3) SVM with all features and with jittering and best *p*_*t*_ threshold, and (4) SVM with the best 35 features and with jittering and best *p*_*t*_ threshold.

**Figure 2:**
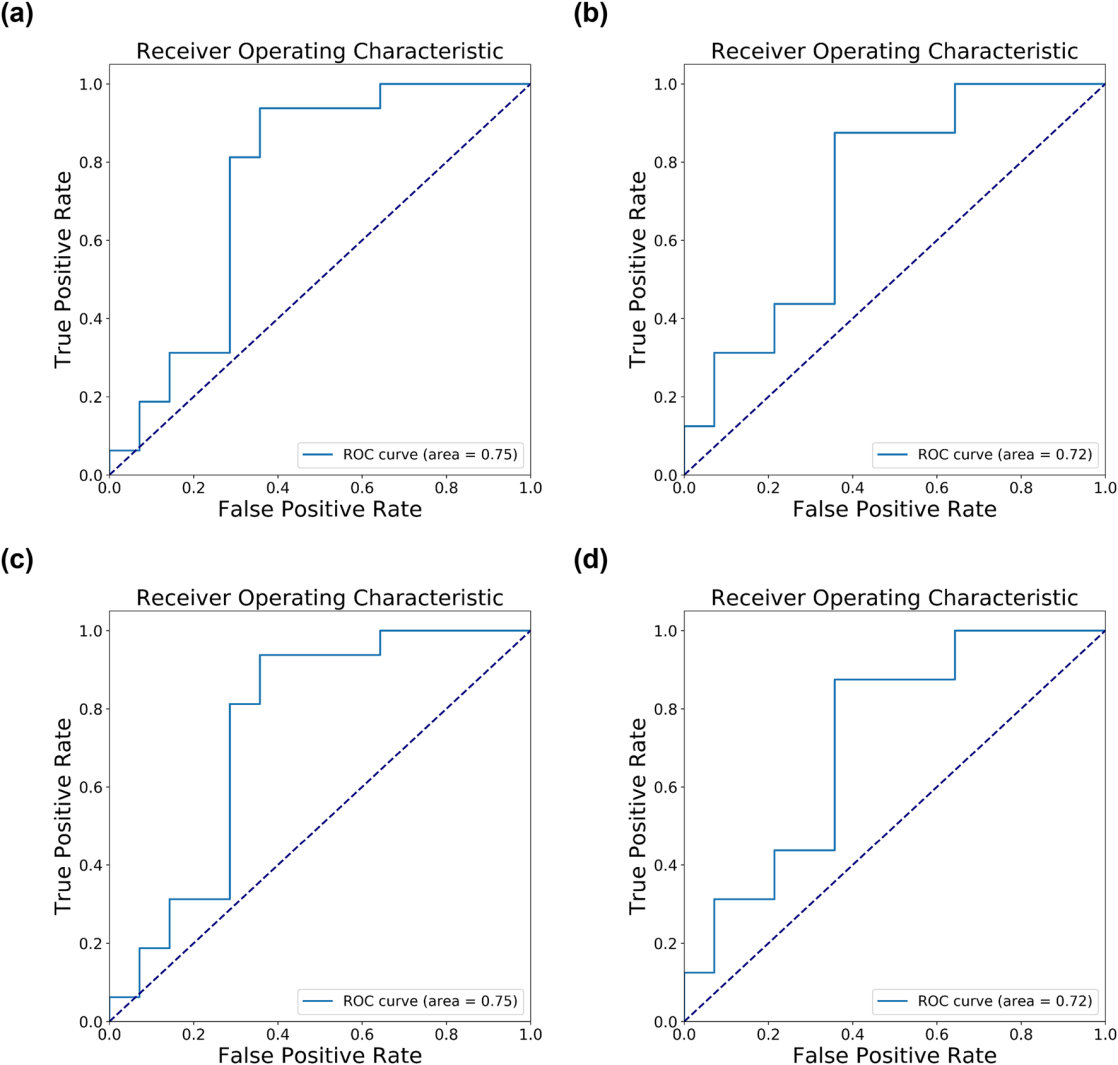
The ROC curve of the test set with transcriptome data only. (**a**) All features (44,608) + SVM (rbf). (**b**) Chi-squared test (35) + SVM (rbf). (**c**) All features (44,608) + SVM (rbf), with noise (*I* = 0.001) and probability threshold = 0.3. (**d**) Chi-squared test (35) + SVM (rbf), with noise (*I* = 0.001) and probability threshold = 0.3.

Results show that feature selection improves the performance as expected; both SVM models with feature selection have higher F1 scores (0.76) than models without feature selection (0.71 and 0.73, Table 2). However, the impact of feature selection is not dramatic as the F1 score improves by at most 0.05, similar to the enhancement in the training set (from an F1 score of 0.7397 without feature selection to an F1 score of 0.7809 with feature selection). Also, improvements that include jittering and the best *p*_*t*_ threshold do not increase the performance as the F1 score of the SVM model with those improvements is identical to that without the improvements in the test set, although we observe higher F1 scores with the improvements in the training set where we observed F1 score of 0.80 with the best *p*_*t*_ threshold and F1 score of 0.78 without the improvement. In terms of AUC under the ROC curve, the best AUC is observed when using all features (AUC of 0.75) while the SVM models with feature selection have slightly lower AUC (0.72, Fig. 2). The modest enhancement in performance by feature selection and other improvements in the test set may be due to the small sample size of the test set. We also examined the performance of our AD classifier with only 19 of the 35 selected probes, which explicitly represents expressed genes with gene symbols (Supplementary Table S5). We observed the greatly increased performance of our AD classifier, which achieved an F1 score of 0.84 (Supplementary Table S6). Interestingly, applying jittering and thresholding did not further improve its performance. It was probably due to the smaller number of selected features that were more representative and informative. So the overfitting issue might be mitigated and thus it is unnecessary to use jittering and thresholding.

**Table 2:** The results on different methods with transcriptome data only.

Feature selection method (number of features) + Classification method	F1 score	Accuracy	Precision	Recall
All features (44,608) + SVM (rbf)	0.7272	0.6000	0.5714	**1.0000**
chi-squared test (35) + SVM (rbf)	**0.7647**	**0.7333**	**0.7222**	0.8125
All features (44,608) + SVM (rbf), with noise (*I* = 0*.*001) and probability threshold = 0.3	0.7111	0.5667	0.5517	**1.0000**
chi-squared test (35) + SVM (rbf), with noise (*I* = 0*.*001) and probability threshold = 0.3	**0.7647**	**0.7333**	**0.7222**	0.8125

The first method trained the model on the original training set without feature selection. The second method performed feature selection by chi-squared test and selected 35 features. For the last two methods, they are similar with the first two methods respectively while the only difference was that they added the noise and changed the probability threshold. The random seed of the noise was 21, which was the best result on this intensity (*I* = 0*.*001).

### Evaluation of the classifier with microbiota data

In addition to the transcriptome data, we have microbiota data from 161 individuals with 366 phylum and genus features, and we build the ML classifier that uses both transcriptome and microbiota data (Fig. 1). Similar to the transcriptome-only classifier, we first perform feature selection on the microbiota features using a training set (n = 131) with the same cross-validation approach and feature selection methods. If using microbiota data only, we observe the best performance in terms of F1 score (0.73) with the SVM approach using 25 microbiota features (Supplementary Table S1). Additionally, we perform feature selection after combining microbiota and transcriptome data and find that 50 microbiota and 35 transcriptome features generate higher F1 scores (Supplementary Table S1). As for the other improvements in the ML model, we use the same probability threshold (*p*_*t*_ = 0.3) and noise level (*I* = 0.001) as ones we used for the transcriptome-only classifier; these thresholds and noise levels also generate the best performance (Supplementary Table S7, S8).

**Figure 3:**
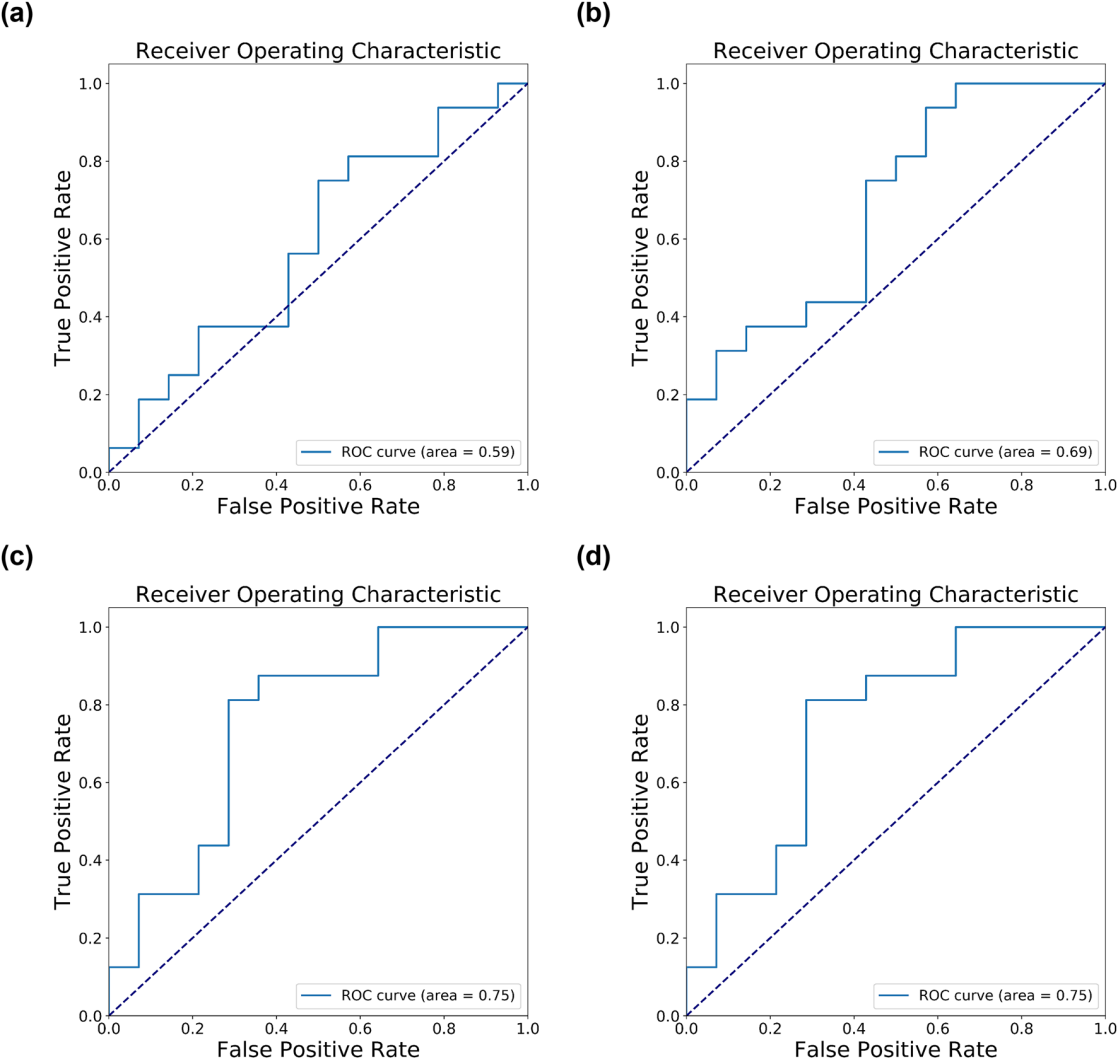
The ROC curve of the test set with microbiota data. (**a**) All features (366) + SVM (rbf). (**b**) Chi-squared test (25) + SVM (rbf). (**c**) Chi-squared test (85) + SVM (rbf). (**d**) Chi-squared test (85) + SVM (rbf), with noise (*I* = 0.001) and probability threshold = 0.3. For panel (**a**,**b**), we only use microbiota data, while for (**c**,**d**) we also include transcriptome data.

Next, we evaluate the prediction ability of the microbiota data on AD using six different classifiers with a test set (n = 30): (1) SVM using all microbiota features, (2) SVM using the best 25 microbiota features, (3) SVM using the best 50 microbiota and 35 transcriptome features, (4) the first, (5) the second, and (6) the third models with *p*_*t*_ and jittering improvements. Results show that the classifiers that combine microbiota and transcriptome data (the third and sixth models) are most accurate in predicting AD, achieving an F1 score of 0.78 (Table 3). The classifiers that use only microbiota data generally have lower accuracy (F1 scores between 0.69 and 0.74) than ones that use both microbiota and transcriptome data. Compared to the previous transcriptome-only classifiers that have the best F1 score of 0.76, the microbiota data marginally improve the classifier performance to an F1 score of 0.78. In terms of area under the ROC curve (AUC), the microbiota data does not improve the performance compared to the transcriptome-only classifiers as the best AUC is identical (0.75) between the classifier that combines microbiota and transcriptome data and the transcriptome-only classifier (Fig. 3). Additionally, we selected 19 transcriptomic features with gene names and 50 microbiota features to train our AD classifier. We found that it did not perform better than using all 35 selected transcriptome features and 50 microbiota features, where it achieved an F1 score of 0.7273 initially and 0.7778 after applying jittering and thresholding (Supplemental Table S9).

**Table 3:** The first and second methods used microbiota data only.

Feature selection method (number of features) + Classification method	F1 score	Accuracy	Precision	Recall
All features (366) + SVM (rbf)	0.7111	0.5667	0.5517	**1.0000**
chi-squared test (25) + SVM (rbf)	0.7442	0.6333	0.5926	**1.0000**
chi-squared test (85) + SVM (rbf)	**0.7778**	**0.7333**	**0.7000**	0.8750
All features (366) + SVM (rbf), with probability threshold = 0.3	0.6957	0.5333	0.5333	**1.0000**
chi-squared test (25) + SVM (rbf), with probability threshold = 0.3	0.7111	0.5667	0.5517	**1.0000**
chi-squared test (85) + SVM (rbf), with noise (*I* = 0*.*001) and probability threshold = 0.3	**0.7778**	**0.7333**	**0.7000**	0.8750

The first method trained the model on the original training set without feature selection. The second method did feature selection by chi-squared test and selected 25 features, while the third method used both transcriptome and microbiota data, and integrated the data using the fourth plan mentioned above, and selected 85 features (35 for transcriptome and 50 for microbiota). For the last three methods, they are similar with the first three methods respectively. The only difference was that they changed the threshold and added noises.

### Top genes selected in the AD classifier

**Figure 4:**
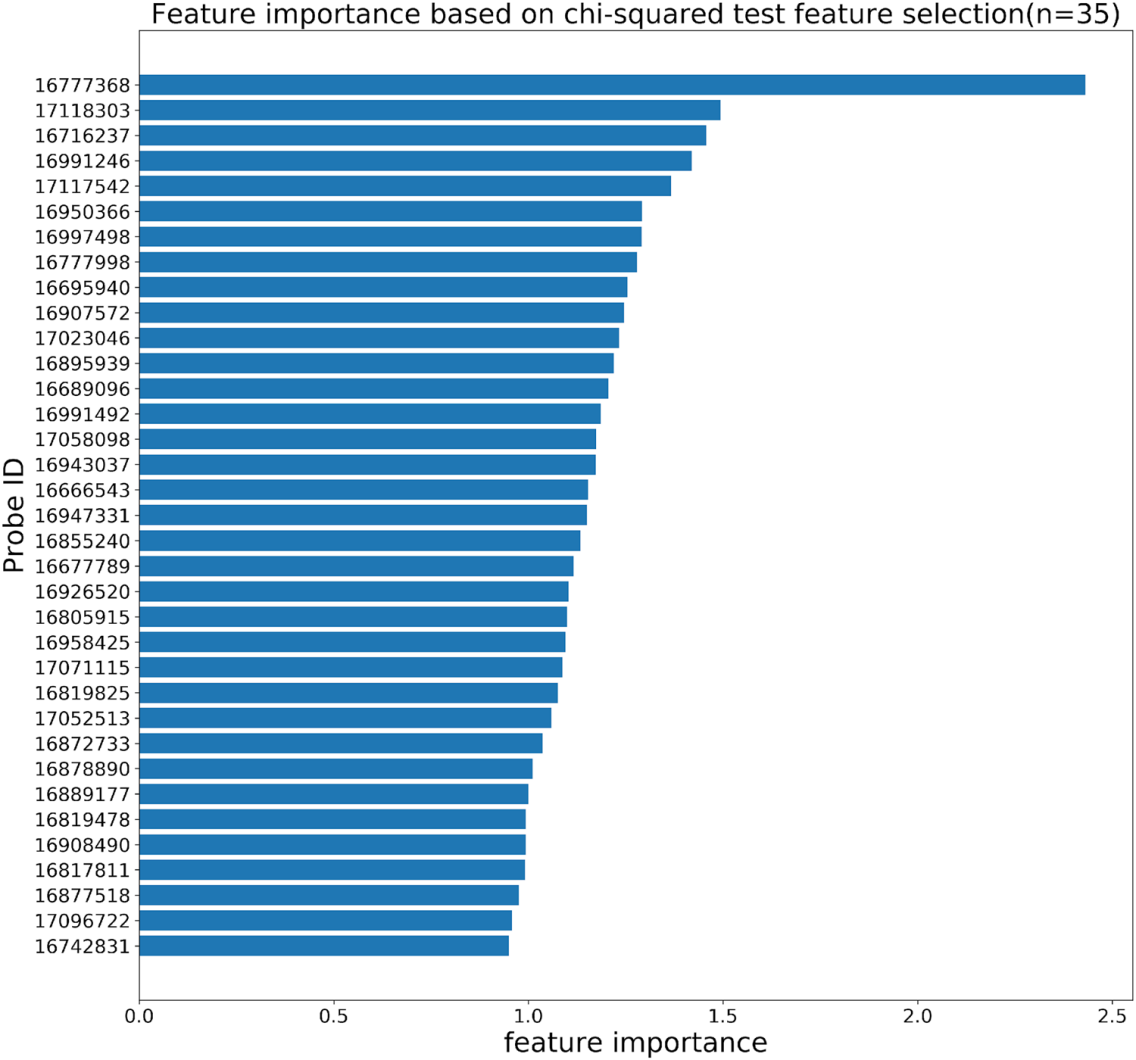
The average feature importance of the top 35 selected probes/genes. See more detailed annotation information in Supplementary Table S5.

Our feature selection algorithm using the transcriptome data identifies 35 features or probes (Fig. 4) that span over 19 unique genes. These genes are selected as they are most informative in predicting AD, which suggests they may be implicated in AD. Hence, we perform a literature search for these 19 genes to examine whether they are known to be related to AD and find a few cases. First, we find that *GRP1* (Probe ID: 16907572) is associated with a type of scaffold protein (Grasp) that potentially influences p53-mediated apoptotic responses in the skin^33^. It is known that apoptosis is a crucial process in the development of AD^34,35^. Second, another gene called *CCL22* (Probe ID: 16819478) is known to play an important role in AD pathogenesis. It encodes chemokine (C–C motif) ligand 22, which is involved in the immunoregulatory and inflammatory processes of T cells. Additionally, *CCL22* is found to be one of the important biomarkers of severity in infantile AD according to a study involving 34 patients^36^. This gene has also been reported to be significantly up-regulated with AD in a high-throughput proteomic assay^37^ and a transcriptomic analysis^38^. Association studies and functional studies further suggest that the mutations in *CCL22* affect the susceptibility to AD in a gain-of-function manner^39^. Lastly, according to a genome-wide association study, four SNPs associated with Alopecia are mapped to our selected gene, *TTC27* (Probe ID: 16878890)^40^. A previous study found that patients with Alopecia have a higher risk for AD^41^. Overall, these examples demonstrate the clinical importance of our selected genes.

### Top microorganisms in microbiota selected in the AD classifier

**Figure 5:**
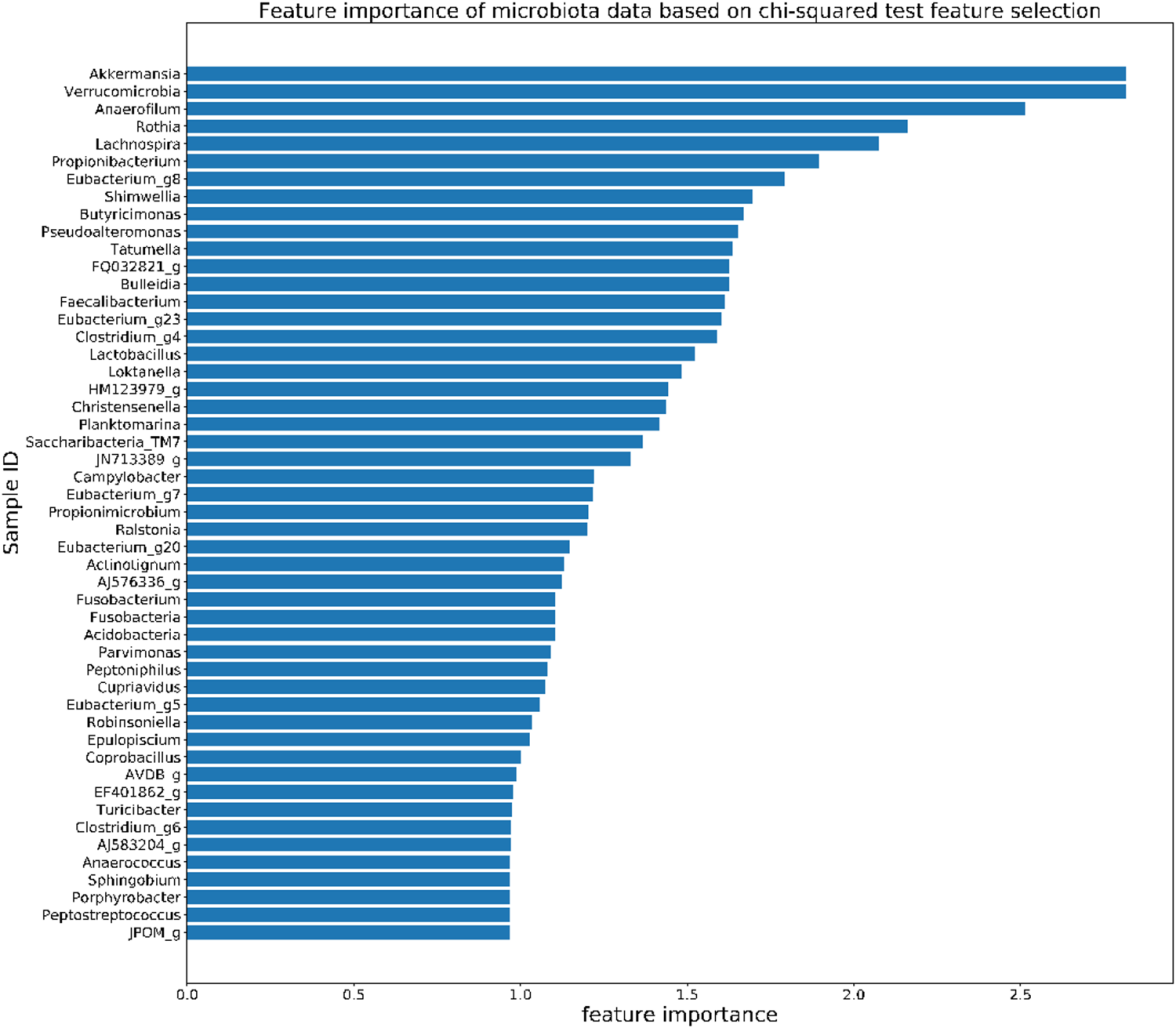
The average feature importance of the top 50 selected microorganisms from the microbiota dataset.

Our feature selection algorithm using the microbiota data and transcriptome data identifies 50 microbiota features (Fig. 5). These microorganisms are chosen to be top predictors for AD, so they may be involved in AD. To validate our findings, we perform a literature search for these 50 kinds of bacteria to look for related studies and supporting evidence. Here are some examples. First, *Akkermanisia* has the highest feature importance in our AD classifier, indicating that the amounts of *Akkermanisia* can affect our prediction the most. A recent study found that the amounts of *Akkermanisia* are high in transient AD patients but low in children with persistent AD^42^. So *Akkermanisia* can be a crucial microbiota indicator for AD. Second, a metagenomic analysis of microbe-derived extracellular vesicles discovered that *Verrucomicrobia*, the bacteria with the second highest feature importance in our AD classifier, had significantly different relative abundances between the AD and control groups and could be used as a novel biomarker for AD diagnosis^43^. Lastly, *Propionibacterium* is ranked the sixth most important microorganism in our AD classifier. It was reported that the relative abundance of *Propionibacterium* is usually reduced and the abundance of *Staphylococcus aureus* is elevated in the skin of AD patients^44^, leading to dysbiosis. Another study observed a dysbiotic status characterized by a reduction of *Propionibacterium* in the gut microbiota of AD patients^45^. And dysbiosis is considered to be an essential driving factor of AD^46,47^. Hence, the selected 50 microbiota features demonstrate the close relationship between gut microbiota and the pathogenesis of AD^48^.

## Discussion

AD is a paradigmatic chronic inflammatory skin disease characterized by complex pathophysiology and a wide spectrum of clinical phenotypes. In particular, the phenotype of AD in early childhood may be influenced by genetic factors and gut microbiota. The purpose of this study was to predict the phenotype of atopic dermatitis in early childhood with transcriptome and microbiota data. Therefore, to understand this diversity, efforts to find new AD endotypes by ML technique using these multi-omics are needed. In this study, we integrated and took the advantage of one of the largest transcriptomic and microbial profiles for AD patients to the best of our knowledge. We developed an AD classifier solely based on transcriptome and microbiota data, which accurately distinguished subjects with AD from healthy individuals. The most accurate classifier selected 35 genes and 50 microbial features (4 phyla and 46 genera) interpreted via a support vector machine classifier, which can automatically classify AD with high precision (0.70) and recall (0.88). Also, among the selected genes/probes used in the AD classifier, we discovered that at least three genes are reported to be directly or indirectly associated with AD. In summary, our classifier represents the first step toward a precise, automated diagnosis of AD and provides important biological insights into the development of the biomarkers of this disease.

Recently, our colleagues have developed an estimated prediction model by multi-omics analyses and realized the importance of transcriptome data^49^; therefore, this study performed the extended analyses with a larger sample size and a different machine learning model for a more precise prediction. Our AD classifier is the first machine learning classifier for this disease based on the transcriptomic and microbial profiles of patients. To diagnose AD, clinicians typically rely on the clinical features of AD^50^. However, the lack of robust objective measures might have negative effects on the assessment of AD^17–19,51,52^. To overcome these challenges, previous studies developed machine learning classifiers for AD diagnosis or severity evaluation based on electronic health records (EHR)^53^, camera photos^54^, or multiphoton tomography^55^. While these approaches may provide an unbiased diagnosis of AD, they are either not highly accurate, achieving F1 scores of 0.67 using EHR^53^ and F1 scores of 0.69 using camera photos^54^, or it may be more inconvenient or expensive to obtain these kinds of data for the ML classifier. With the development of high-throughput microarray and sequencing technologies, it may be and is likely to be cheaper in near future to obtain transcriptome and microbiota data. Another advantage of our ML classifier is that it does not require patients to be present in the testing sites or hospitals as they can send their samples to the labs to generate transcriptome and microbiota data and our classifier can predict the risk of AD based on the data. Thus, our classifier enables the convenient, efficient, and cost-effective diagnosis of AD as well as improving the accessibility of medical resources for patients.

In further enrichment analysis using Enrichr (https://maayanlab.cloud/Enrichr/)^56^ based on the featured genes, interleukin-7 (IL-7) interactions in the immune response pathway (*P* = 0.032, Supplementary Table S10) was enriched. IL-7 is a critical cytokine for the development of the group 2 innate lymphoid cells (ILC2s), which are involved in allergic diseases including AD^57^. In addition, several inflammation-related processes (for instance, lymphocyte, chemokine, neutrophil, *P* < 0.05) were enriched in gene ontology observation. Inflammatory responses associated with lymphocyte, chemokine, and neutrophil are important in AD mediated by CD4+ T cells^58^. These results suggest that featured genes in this study might be potentially valuable for AD diagnosis.

There are a few study limitations. First, the sample size of our dataset is relatively small. As we only used 161 samples recruited from the birth cohort follow-up group and outpatients group, it could cause overfitting and biases when training the ML classifier. To address this issue, we applied nested cross-validation to perform feature selection and model training. We also introduced jittering to add a small amount of artificial noise into the data to reduce overfitting. We showed that we successfully controlled the biases and overfitting as our classifier performed well on the independent test set. Our study also had the limited ability to assess the benefits of adding microbiota data to the ML classifier as we observed the marginal improvement in prediction accuracy, possibly due to the small sample size of the test set when we combined transcriptome and microbiota data. Second, since our subjects from the birth cohort follow-up group are general population and usually considered to have mild severity of AD, there is a possibility that the results may differ from those in the severe patient group. Therefore, to validate and improve the ML classifier and to more accurately assess its performance, further studies in a larger sample size and in an independent cohort are required. Third, age should be considered as a confounding factor to affect the gene expression and gut microbiota in infants through developmental stages. Fourth, it is known that the gut microbiota in infancy is largely unstable, but gradually stabilize as it grows during childhood^59^. However, this study analyzed the gut microbiota in infants at ages from 6 to 72 months, without serial microbiome data of the subjects, for the prediction of AD. Therefore, further studies, including a replication study in serial paired subjects, are needed to resolve the differences or changes of the prediction during aging. The strengths of our study could be an application of non-invasive gut epithelial cells from fecal specimens and a possibility to apply to early prediction for AD patients with mild severity and the general population. In addition, to address the issue of no validation, we created an independent test set from the original dataset and demonstrated the accuracy of our classifier, which could serve as the independent cohort.

In this study, we developed an accurate and automated machine learning pipeline for atopic dermatitis classification. This pipeline can not only be used to predict this skin disease but also be generalized to classify other diseases based on transcriptome and microbiota data. It could assist clinicians in diagnosing and assessing diseases and providing timely treatment to patients. It could also provide new endotypes by performing further research. In addition, we demonstrated the utility of combining genomics and cutting-edge artificial intelligence (AI) technologies like machine learning to detect diseases or identify biomarkers. We expect that the increasing availability of genomics and AI technologies would improve the effectiveness and efficiency of medical diagnosis.

## Supplementary Information

Supplementary Information.

## Data Availability

The data supporting the findings of this study are included within the article.
