# Supplementary material for: Accurate diagnosis of atopic dermatitis by combining transcriptome and microbiota data with supervised machine learning
Source: Sci Rep. 2022 Jan 7;12:290. doi: 10.1038/s41598-021-04373-7 (PMC8741793; doi:10.1038/s41598-021-04373-7)
Supplement: Supplementary file 1 — Supplementary Information. [file 41598_2021_4373_MOESM1_ESM.docx]

# Supplementary Methods

## Feature selection

We tested five feature selection methods: RFE-LR, RFE-SVM, RFE-RFC, Chi-square Test, and RFC, each of which employs plan A and plan B. Given the result, we assigned plan A for RFE-LR and RFC, and plan B for RFE-RFC, RFE-SVM and chi-squared test, as it can make these methods achieve higher performance (Supplementary Table S11). After that, we tested each method with multiple feature numbers to select the best feature selection models (Notes: Although we gave the best performance with certain feature numbers, the results were not used to choose the best feature numbers). And the results are shown in Supplementary Figure S3, Table S12, S13. To clarify, we actually tested every feature numbers ranging from 10 to 180 and the charts shown below are just a few of them. And by comparing the best results from each method, we chose chi-squared test using plan B as the feature selection method for our task. Next we should determine the best number of features. We came up with three methods to undertake this task. (1) For the training set, evaluate the performance of different feature numbers on the test set of the outer CV (the validation set), then select the best one. The problem is that due to the insufficiency of data and large features, the model is unstable. To get a reliable result for comparison, we have to take a lot of random seeds for outer CV then average them. However, the results are too close to choose a best on. (2) Use the whole training set to do feature selection. The criterion is the performance on the outer CV test set. The problem is that it suffers from data leak, because the test set is also used for feature selection. **(3)** Calculate feature importance for each training set in outer CV, rank the average feature importance, and choose the top *n* features based on a criterion. It is the method we chose. We defined that a feature is valid if its average feature importance is larger than a threshold. In this case, we set the threshold as 0.95. After the selection, we got 35 features, which meant that we chose 35 as the feature number to be selected in the chi-squared test method. The average feature importance is shown in Fig. 4.

##

## Model selection

Until now, we have settled on the feature selection method. The following step is the classification. We picked some classical classification methods to evaluate their performance using the data after the feature selection step. That is to say, every sample has only 35 features selected from 44608 features. This great reduction not only improved the accuracy in classification, but also accelerated the algorithm. We carefully selected 4 classification models: Logistic Regression (LR), Support Vector Machine (SVM), Random Forest Classifier (RFC), and XGBoost. The first two methods are quite classical and effective in machine learning. RFC is based on the theory of decision tree, while XGBoost is an advanced gradient boosting tree algorithm that performed successfully in various machine learning scenes. We should be noticed that different feature selection methods and the numbers of features they selected might refer to different best training models. Supplementary Table S2 showed that SVM with rbf kernel was the best classification model, outperforming other methods with a large margin. It might seem surprising at first because methods such as XGBoost generally reached high classification accuracy in many cases. However, after analyzing the results, we found that XGBoost had severe overfitting problems in this task even if adjusting hyperparameters to mitigate this situation. In small sample problems, relatively naive methods probably get better outcomes. According to Supplementary Table S3[,](#_3tbugp1) using probability thresholds = 0.3 improves the performance a little (from 0.7564 to 0.8040). For adding noises, we used different intensities and ran the algorithms. We added the noises before feature selection, tested the performance with 50 random seeds and chose the best one. When the noise increases, the average f1 scores will decrease, while the generalization ability will be enhanced. However, too large noise will lead to unstable models. So we chose 0.001 as the intensity of noise (Supplementary Table S4).

## Integration of microbiota data

The experiments above only used transcriptome data. In data integration, we still firstly did prepossessing on microbiota data, which included filling the lost values and standardizing the data. For the following steps, we introduced four plans as followed:

1. Use microbiota data only

2. Use transcriptome data only

3. Concatenate the data first, then do feature selection and train the model

4. Select the features on different datasets separately, concatenate the selected datasets, and train the model

The results of these four plans were shown in Supplementary Table [S1.](#_3tbugp1) For plan 4, we selected 50 features in microbiota data. However, the optimal number of microbiota features in plan 1 was 25. The difference was because in plan 4, we optimized the combined f1 score to select the feature numbers, which meant we should take the prediction ability of transcriptome data into account. In general, we concluded that microbiota data might help with the prediction (although it seemed useless according to the results). However, it was hard to choose the right number of features due to the small size of the dataset. Besides, we also tested the integration results with different thresholds and noises (Supplementary Table S7, S8[).](#_3tbugp1) It turned out that the best threshold was also 0.3. As for the noises, although the noise intensity of 0.0001 had the best Avg. f1 score of 0.7883 while the intensity of 0.003 had the best f1 score of 0.8005. We need to consider both evaluation metrics as mentioned before. Therefore, we also chose 0.001 as the intensity of the noise. The reason we only add noises to transcriptome data is that microbiota data contain many zeros, so that adding noises to microbiota data will affect the results greatly.

**Supplementary Figures**

**Supplementary Figure S1.** Feature selection methods. (a) Plan A includes a nested cross-validation. (b) Plan B only applies the outer CV approach.

**Supplementary Figure S2.** Two examples to illustrate Plan A and plan B. Two examples used the same outer CV training set. (a) choose *m* = 4, so select 4 features in each inner CV fold. After that, calculate the overlap features *n_A_*, which is 2; (b) choose *n_B_* = 3, which means directly select 3 features in the same outer CV training set as Plan A.

**Supplementary Figure S3.** The performance of three feature selection methods. We show the F1 scores of three feature selection methods when selecting 5 to 150 features with a step size 5. The interval between two points is five features.

**Supplementary Tables**

**Supplementary Table S1.** Performance of integrating microbiota data with four different strategies

| Plan | Number of features | Average F1 score | Standard deviation of F1 score |
| --- | --- | --- | --- |
| (1) | 25 | 0.7255 | 0.0405 |
| (2) | 35 | 0.7809 | 0.0483 |
| (3) | 85 | 0.7698 | 0.0322 |
| (4) | 85^1^ | **0.7883** | 0.0385 |

The numbers of features shown in the table had been optimized through the comparison methods mentioned in feature selection and classification parts. ^1^: 85=35+50, which meant 35 features in transcriptome data and 50 features in microbiota data. (Settings: feature selection method: chi-squared test, training model: SVM (rbf))

**Supplementary Table S2.** Model Selection with different methods

| Model | Average F1 score | Standard deviation of F1 score |
| --- | --- | --- |
| LR | 0.7598 | 0.0393 |
| SVM (linear) | 0.7598 | 0.0194 |
| SVM (rbf) | **0.7809** | 0.0483 |
| RFC | 0.7149 | 0.0596 |
| XGBoost | 0.7145 | 0.0430 |

SVM (linear) and SVM (rbf) indicate the kernel functions in SVM algorithm. Evaluate the performance by averaging the f1 scores on outer CV test sets. Feature selection method: chi-squared test, feature number: 35.

**Supplementary Table S3.** Performance of different thresholds

| Threshold | Average F1 score | Standard deviation of F1 score |
| --- | --- | --- |
| 0.3 | **0.8040** | 0.0359 |
| 0.4 | 0.7679 | 0.0132 |
| 0.5 | 0.7564 | 0.0211 |
| 0.6 | 0.6942 | 0.0582 |
| 0.7 | 0.6148 | 0.0371 |

Performance of different thresholds from 0.3 to 0.7 and the results on outer CV test sets f1 scores. Settings: feature selection: chi-squared test, number of features: 35, training model: SVM (rbf).

**Supplementary Table S4.** Performance of different intensities of noises from 0.001 to 0.1

| Intensity | Avg. F1 score | Best F1 score (seed) | Standard deviation of Avg. F1 score |
| --- | --- | --- | --- |
| 0.001 | **0.7827** | 0.7938 (21) | 0.0413 |
| 0.003 | 0.7735 | 0.7965 (31) | 0.0360 |
| 0.005 | 0.7657 | 0.7926 (5) | 0.0340 |
| 0.01 | 0.7560 | 0.7932 (47) | 0.0418 |
| 0.1 | 0.6689 | 0.7471 (29) | 0.0755 |

Settings: feature selection method: chi-squared test, number of features: 35, training model: SVM (rbf).

**Supplementary Table S5.** Annotation information of 35 gene features

| ProbeID | Gene_Symbol | mRNA Accession | Chromosome | Start | Stop |
| --- | --- | --- | --- | --- | --- |
| 16777368 | LINC00621 | XR_914784 | chr13 | 23429412 | 23490508 |
| 17118303 |  |  | chr7 | 94058513 | 94060553 |
| 16716237 |  | ENST00000363383 | chr10 | 88800995 | 88801098 |
| 16991246 |  |  | chr5 | 151041996 | 151043043 |
| 17117542 |  | BC020337 | chr11 | 65266414 | 65278498 |
| 16950366 | GRM7-AS1 | NR_046606 | chr3 | 7561428 | 7602020 |
| 16997498 | SCAMP1-AS1 | NR_105014 | chr5 | 77638189 | 77656331 |
| 16777998 |  | NONHSAT033112 | chr13 | 33337576 | 33337690 |
| 16695940 | LOC105371590 | XR_918028 | chr1 | 166445009 | 166459276 |
| 16907572 | GPR1 | NM_001098199 | chr2 | 207040040 | 207082771 |
| 17023046 |  | ENST00000415736 | chr6 | 117772293 | 117774688 |
| 16895939 |  | ENST00000363133 | chr2 | 27698361 | 27698462 |
| 16689096 |  | NONHSAT004246 | chr1 | 85900858 | 85900967 |
| 16991492 | PPP1R2P3 | NR_038443 | chr5 | 156277549 | 156279539 |
| 17058098 | LOC100287834 | NR_028349 | chr7 | 62856718 | 62859419 |
| 16943037 |  | NONHSAT090753 | chr3 | 96199795 | 96232359 |
| 16666543 |  | NONHSAT004126 | chr1 | 81717474 | 81717781 |
| 16947331 |  | NONHSAT092876 | chr3 | 156735131 | 156735485 |
| 16855240 |  | NONHSAT059255 | chr18 | 47397925 | 47398036 |
| 16677789 | BROX | NM_001288579 | chr1 | 222885895 | 222908538 |
| 16926520 | LOC105372836 | XM_011508909 | chr21 | 46475931 | 46488975 |
| 16805915 | NF1P2 | NR_028506 | chr15 | 22133181 | 22145802 |
| 16958425 |  | ENST00000516833 | chr3 | 124777148 | 124777282 |
| 17071115 | LOC101927039 | XR_242407 | chr8 | 97247655 | 97250262 |
| 16819825 |  | ENST00000563151 | chr16 | 66584360 | 66584470 |
| 17052513 | MGAM2 | NM_001293626 | chr7 | 141811549 | 141843783 |
| 16872733 |  | NONHSAT066522 | chr19 | 42648206 | 42648277 |
| 16878890 | TTC27 | NM_001193509 | chr2 | 32853087 | 33046118 |
| 16889177 |  | ENST00000489557 | chr2 | 200190033 | 200194085 |
| 16819478 | CCL22 | NM_002990 | chr16 | 57392684 | 57400102 |
| 16908490 | CRYBA2 | NM_057093 | chr2 | 219854911 | 219858143 |
| 16817811 | PPP4C | NM_001303503 | chr16 | 30087311 | 30096698 |
| 16877518 | MIR4757 | NR_039914 | chr2 | 19548190 | 19548266 |
| 17096722 | OR13C2 | BC136824 | chr9 | 107366924 | 107367951 |
| 16742831 | SNORA70E | NR_033347 | chr11 | 82752506 | 82752640 |

**Supplementary Table S6.** Classification performance with only 19 probes with gene names

| Selected features + Classification method | F1 score | Accuracy | Precision | Recall | AUC |
| --- | --- | --- | --- | --- | --- |
| 19 genes + SVM (rbf) | 0.8387 | 0.8333 | 0.8667 | 0.8125 | 0.8348 |
| 19 genes + SVM (rbf), with noise (*I* = 0*.*001) and probability threshold = 0.3 | 0.7879 | 0.7667 | 0.7647 | 0.8125 | 0.7634 |

**Supplementary Table S7.** Performance of different thresholds

| Threshold | Average F1 score | Standard deviation of F1 score |
| --- | --- | --- |
| 0.2 | 0.7647 | 0.0425 |
| 0.3 | **0.7866** | 0.0333 |
| 0.4 | 0.7858 | 0.0370 |
| 0.5 | 0.7792 | 0.0145 |
| 0.6 | 0.7191 | 0.0342 |
| 0.7 | 0.6582 | 0.1027 |
| 0.8 | 0.5949 | 0.0458 |

Performance of different thresholds from 0.2 to 0.8 and the results on outer CV test sets f1 scores. Settings: feature selection method: chi-squared test, number of features: 35 for transcriptome data and 50 for microbiota data, training model: SVM (rbf).

**Supplementary Table S8.** Performance of different intensities of noises from 0.0001 to 0.1

| Intensity | Avg. F1 score | Best F1 score (seed) | Standard deviation of Avg. F1 score |
| --- | --- | --- | --- |
| 0.0001 | **0.7883** | 0.7983 (0) | 0.0345 |
| 0.001 | 0.7867 | 0.7953 (18) | 0.0340 |
| 0.003 | 0.7778 | **0.8005 (31)** | 0.0319 |
| 0.005 | 0.7758 | 0.7982 (18) | 0.0322 |
| 0.01 | 0.7695 | 0.8003 (37) | 0.0390 |
| 0.1 | 0.6966 | 0.7758 (37) | 0.0685 |

Settings: feature selection method: chi-squared test, number of features: 35 for transcriptome data and 50 for microbiota data, training model: SVM (rbf). The noises are added to transcriptome data.

**Supplementary Table S9.** Classification performance with 19 transcriptomic features with gene names and 50 microbiota features

| Selected features + Classification method | F1 score | Accuracy | Precision | Recall | AUC |
| --- | --- | --- | --- | --- | --- |
| 19 genes + 50 microbiota features + SVM (rbf) | 0.7778 | 0.7333 | 0.7000 | 0.8750 | 0.7232 |
| 19 genes + 50 microbiota features + SVM (rbf), with noise (*I* = 0*.*001) and probability threshold = 0.3 | 0.7272 | 0.7000 | 0.7059 | 0.7500 | 0.6964 |

**Supplementary Table S10.** Enrichment analysis of featured genes

| **Pathway** | ***P*-value** | **Platform** |
| --- | --- | --- |
| Interleukin-7 interactions in immune response | 0.032 | BioPlanet 2019 |
| Galactose metabolism | 0.029 | KEGG 2019 |
| Starch and sucrose metabolism | 0.034 | KEGG 2019 |
| Carbohydrate digestion and absorption | 0.041 | KEGG 2019 |
|  |  |  |
| **Gene Ontology** | ***P*-value** | **Platform** |
| regulation of double-strand break repair via homologous recombination (GO:0010569) | 0.025 | GO biological process 2018 |
| regulation of DNA recombination (GO:0000018) | 0.031 | GO biological process 2018 |
| regulation of double-strand break repair (GO:2000779) | 0.032 | GO biological process 2018 |
| regulation of phosphoprotein phosphatase activity (GO:0043666) | 0.033 | GO biological process 2018 |
| regulation of protein dephosphorylation (GO:0035304) | 0.037 | GO biological process 2018 |
| monocyte chemotaxis (GO:0002548) | 0.038 | GO biological process 2018 |
| lymphocyte chemotaxis (GO:0048247) | 0.041 | GO biological process 2018 |
| chemokine-mediated signaling pathway (GO:0070098) | 0.048 | GO biological process 2018 |
| neutrophil chemotaxis (GO:0030593) | 0.049 | GO biological process 2018 |

**Supplementary Table S11.** The experiments on choosing Plan A or Plan B for different methods.

| Method | *n_A_* | *n_B_* | F1 score of Plan A | Standard deviation of F1 score of Plan A | F1 score of Plan B | Standard deviation of F1 score of Plan B |
| --- | --- | --- | --- | --- | --- | --- |
| RFE-LR | 87 | 90 | **0.6834** | 0.0719 | 0.6472 | 0.0646 |
| RFE-SVM | 71 | 70 | 0.7007 | 0.0617 | **0.7270** | 0.0481 |
| RFE-RFC | 220 | 220 | 0.6926 | 0.0410 | **0.7026** | 0.0778 |
| Chi-squared Test | 53 | 50 | 0.6906 | 0.0657 | **0.7539** | 0.0855 |
| RFC | 155 | 150 | **0.6788** | 0.0454 | 0.6331 | 0.0464 |

**Supplementary Table S12.** RFE-LR

| n_A_ | Best F1 score(seed) | Avg. F1 score | Standard deviation of Avg. F1 score |
| --- | --- | --- | --- |
| 32 | 0.7078(0) | 0.6836 | 0.0716 |
| 42 | 0.7197(7) | **0.6849** | 0.0673 |
| 52 | 0.7185(0) | 0.6753 | 0.0610 |
| 63 | 0.7128(0) | 0.6715 | 0.0727 |
| 74 | 0.7041(0) | 0.6671 | 0.0694 |
| 87 | 0.7115(1) | 0.6834 | 0.0719 |
| 100 | 0.7239(0) | 0.6823 | 0.0683 |
| 113 | 0.7264(0) | 0.6823 | 0.0606 |
| 127 | 0.6974(1) | 0.6806 | 0.0680 |
| 141 | 0.7040(6) | 0.6847 | 0.0627 |
| 156 | 0.7220(2) | 0.6822 | 0.0683 |

**Supplementary Table S13.** RFE-RFC

| n_A_ | Best F1 score(seed) | Avg. F1 score | Standard deviation of Avg. F1 score |
| --- | --- | --- | --- |
| 33 | 0.6466(2) | 0.6308 | 0.0485 |
| 43 | 0.6379(4) | 0.6283 | 0.0579 |
| 53 | 0.6462(2) | 0.6291 | 0.0521 |
| 63 | 0.6566(4) | 0.6405 | 0.0560 |
| 73 | 0.6579(6) | 0.6454 | 0.0639 |
| 84 | 0.6531(7) | 0.6307 | 0.0515 |
| 94 | 0.6383(7) | 0.6257 | 0.0396 |
| 104 | 0.6646(1) | 0.6369 | 0.0396 |
| 129 | 0.6629(9) | 0.6488 | 0.0365 |
| 155 | **0.6957(6)** | **0.6788** | 0.0454 |
